# Analysis of Temporal and Spatial Changes in Ecological Environment Quality on Changxing Island Using an Optimized Remote Sensing Ecological Index

**DOI:** 10.3390/s25061791

**Published:** 2025-03-13

**Authors:** Yuanyi Zhu, Yingzi Hou, Fangxiong Wang, Haomiao Yu, Zhiying Liao, Qiao Yu, Jianfeng Zhu

**Affiliations:** 1 School of Geographical Sciences, Liaoning Normal University, No. 850, Huanghe Road, Dalian 116029, China; zhuyyi0215@ldy.edu.rs (Y.Z.); hyz@lnnu.edu.cn (Y.H.); yhmhhxx@dlmu.edu.cn (H.Y.); gis_lzy@163.com (Z.L.); 18741527978@163.com (Q.Y.); zjf014@lnnu.edu.cn (J.Z.); 2 Liaoning Provincial Key Laboratory of Physical Geography and Geomatics, Liaoning Normal University, Street 15, Dalian 116029, China

**Keywords:** island, land cover change classification, coastline, ecological quality, Google Earth Engine

## Abstract

In light of global climate change and accelerated urbanization, preserving and restoring island ecosystems has become critically important. This study focuses on Changxing Island in Dalian, China, evaluating the quality of its ecological environment. The research aims to quantify ecological changes since 2000, with an emphasis on land use transformations, coastline evolution, and the driving factors behind these changes. Using the Google Earth Engine (GEE) platform and remote sensing technology, an island remote sensing ecological index (IRSEI) was developed. The development of the IRSEI was grounded in several key ecological parameters, including the normalized difference vegetation index (NDVI), wetness index (WET), land surface temperature index (LST), multiband drought stress index (M-NDBSI), and land use intensity index (LUI). The research results show that, since 2002, land use types on Changxing Island have undergone significant changes, with a notable decrease in arable land and a significant increase in built-up areas, reflecting the ongoing urbanization process. With respect to coastline changes, the total coastline length of Changxing Island steadily increased from 2002 to 2022, with an average annual growth rate of 2.15 km. This change was driven mainly by reclamation and infrastructure construction. The IRSEI analysis further revealed a clear deterioration in the quality of the ecological environment of Changxing Island during the study period. The proportion of excellent ecological area decreased from 39.3% in 2002 to 8.89% in 2022, whereas the areas classified as poor and very poor increased to 56.23 km^2^ and 129.84 km^2^, both of which set new historical records. These findings suggest that, as urbanization and coastline development intensify, the ecosystem of Changxing Island is at significant risk of degradation. The optimized IRSEI effectively captured the ecological environment quality of the island, improved the long-term stability of the index, and adequately met the requirements for large-scale and long-term ecological environment quality monitoring.

## 1. Introduction

In recent decades, coastal areas have undergone rapid urbanization and economic development, leading to significant changes in land use and the ecological environment [1]. These changes were driven in part by global climate change, population growth, changes in land use, human activities, and terrestrial pollution [2]. Extreme climate variability, population surges, irrational human behaviours, changes in terrain and landforms, accelerated urbanization, and the overexploitation of natural resources have had significant impacts on the stability of ecosystems and their service functions [3,4,5,6].

Related academic research suggests that there is a positive relationship between improved ecosystem services and widespread vegetation coverage, land use practices, and climate change [7]. Komolafe and Rosazlina (2022) identified urbanization as the main driver of changes in land use and land cover on Bintang Island between 2010 and 2021, leading to increased surface temperatures and altered vegetation indices [8]. Changes in the ecosystem state in the Amazon Basin floodplain were used to determine the effects of flood variations on biodiversity and ecosystem services [9]. Land use and land cover change (LULC) has a significant impact on ecosystem services, particularly in terms of water resource changes [10]. Research indicates that variations in surface temperatures are linked to urbanization, shifts in land cover, and climate fluctuations [11]. Researchers have used nearly 30 years of Landsat imagery to obtain coastline and land reclamation information and applied ecological risk indices to explore the ecological risk characteristics of land use changes in reclamation areas [12]. Between 2003 and 2014, the southwestern coastline of Bahrain Island retreated at a rate of 5 m per year, significantly exceeding the modelled retreat rate derived from local tidal station data [13]. Remote sensing analysis from 2000 to 2020 revealed a 462.57% increase in impermeable surfaces on Haitan Island, accompanied by declines in arable land and water areas, with urbanization and tourism development driving significant increases in landscape fragmentation and spatial heterogeneity [14]. In recent years, the accelerated construction of transportation links between islands and the mainland has led to increasing human interference in island ecosystems. Xie et al. (2018) assessed the ecological vulnerability of Zhujiajian Island via the Island Ecological Vulnerability Index (IEVI) and simulated changes in tourism and land use over 20 years [15]. On the basis of Landsat remote sensing data, Xi et al., 2021 analysed spatiotemporal changes in the normalized difference vegetation index (NDVI) to explore ecosystem service value (ESV) trends in 12 island counties (cities) in China. The study revealed a continuous decline in the NDVI of island counties from 1990 to 2018, and the impact of different land cover types on the ecosystem service value was evaluated [16]. In recent years, changes in island ecological environments, especially wetland ecosystems, have garnered significant attention. Remote sensing data have been used to study the spatiotemporal evolution and ecological risk of coastal wetlands on Hainan Island, highlighting the impacts of human activities and climate change [17]. Li et al., 2023 utilized LULC change data to develop a landscape ecological risk assessment model to study the ecological risks on Zhoushan Island, Zhejiang. The study, which spanned from 2000 to 2020, revealed significant spatiotemporal variations in landscape ecological risk, with ecological risk levels undergoing notable changes as built−up areas expanded [18]. An assessment model consisting of 15 indicators was proposed and, with the progression of urbanization, the ecological vulnerability of Chongming Island was found to exhibit spatial distribution differences [19]. Although existing studies provide important references for island ecological monitoring, most focused on single factors or case studies of specific islands, overlooking the multidimensional changes in ecosystems, especially in terms of the comprehensive assessment of various ecological factors in remote sensing data analysis.

The Remote Sensing−derived Ecological Index (RSEI) combines four key factors greenness, humidity, dryness, and temperature−through a principal component analysis (PCA). These factors form the first principal component (PC1), providing a comprehensive evaluation index [20]. Through the comprehensive assessment of the RSEI, the evolution patterns of ecological issues such as reduced vegetation cover, land degradation, and water pollution can be revealed, and the combined impact of natural and human factors on the ecological environment can be assessed [21]. The RSEI is capable of providing reliable data support for large−scale ecological monitoring and can aid in developing effective environmental management and conservation strategies [22,23]. Although RSEI has advantages in environmental quality assessment, building RSEI−based models in traditional remote sensing software, especially for long−time series and large−scale applications, is very complex and time−consuming.

Remote sensing technology, with short observation cycles, high cost efficiency, and multisource data advantages, offers extensive data and technical support for monitoring island land cover [24]. Researchers focusing on islands have used time series of Landsat 8 imagery, the Google Earth Engine (GEE) platform, and the random forest algorithm to successfully map tidal flats in eastern coastal China, with an overall accuracy of 94.4% [25]. The GEE is a cloud−based platform that provides powerful tools and resources for large−scale geospatial data analysis [26]. The GEE excels in handling and analysing large remote sensing datasets, particularly in processing Landsat and Sentinel−2A imagery as well as digital elevation data [27]. Additionally, the GEE can be applied in land cover mapping, allowing users to perform a range of tasks, such as data preprocessing, image classification, and result validation [28]. Compared with traditional remote sensing data acquisition methods and data processing software, the GEE has several advantages, such as extensive historical data access, stable operational performance, high computational efficiency, a user−friendly interface, and excellent cost effectiveness [29]. Moreover, researchers used Landsat 8, Landsat ETM+, and TM data to analyse coastline morphology changes in the northern Bay of Bengal from 1989 to 2018 via the GEE platform. The study revealed significant changes in the coastline over these 30 years, with the land area increasing by 1.15%; some new islands formed, whereas some old islands disappeared [30].

In the coastal regions of China, the scale of land reclamation projects in Bohai Bay ranks among the largest in the country, and their impact on the surrounding ecological environment and economic activities cannot be ignored [31]. Located in a key position in the sea area, the Liaoning Changxing Island Port Industrial Zone is the core area of the coastal economic development strategy, with the objective of promoting regional economic growth and industrial upgrading. As an indispensable part of Dalian Port, Changxing Island plays an extremely critical role in driving local economic progress and attracting investment. In this context, balancing environmental protection with regional economic development has become a key issue on Changxing Island. As a result, scientific monitoring of dynamic changes in the ecological environment is needed to provide decision−making support for future sustainable development. Therefore, the objectives of this study include the following: (1) using the GEE platform to integrate multiple remote sensing datasets (such as Landsat and Sentinel−2A) to construct the island remote sensing ecological index (IRSEI); (2) quantifying spatiotemporal changes in environmental quality on Changxing Island since 2002; (3) analysing the impacts of land use and coastline dynamics on ecosystem services; and (4) exploring spatial differences in the characteristics of island ecosystems. This approach provides an effective and economical method for evaluating the quality of the island ecological environment on the basis of the GEE and establishing scientifically sound strategies for ecological management and sustainable development in areas with intense development.

## 2. Materials and Methods

### 2.1. Study Area

**Figure 1:**
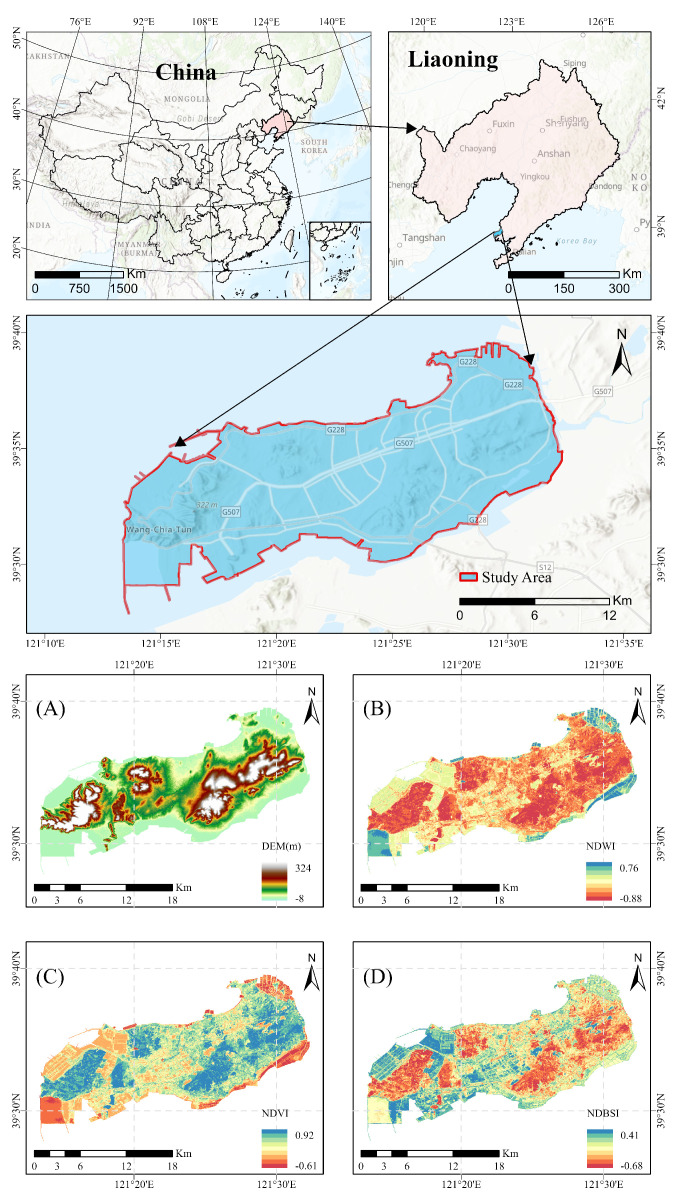
Location of Changxing Island. (**A**) DEM (Digital Elevation Model), (**B**) NDWI (Normalized Difference Vegetation Index), (**C**) NDVI (Normalized difference vegetation index), (**D**) NDBSI (Normalized difference drought index).

Changxing Island is situated in the west−central part of the Liaodong Peninsula, bordering Wafangdian city in Dalian to the west. Surrounded by the Bohai Sea, it is linked to the mainland by two bridges, with the closest point to the mainland on the strait−facing side just 358 m off the coast. The total area of the island is 252.5 km^2^, located between 120° and 121° E longitude and 31° and 39° N latitude. It is located to the south of Dalian and to the north of the central Liaoning city cluster, including Anshan and Shenyang. The island is strategically positioned 292 km from Shenyang, 83 km from Dalian, 59 nautical miles from Lüshunkou, 170 nautical miles from Tianjin Port, 339 nautical miles from Incheon Port in South Korea, and 646 nautical miles from Nagasaki Port in Japan (Figure 1). Figure 1 presents various ecological indicators for Changxing Island. Panel A shows the digital elevation model (DEM), which reflects the island’s topographic variations; Panel B displays the normalized difference water index (NDWI), which reflects the distribution of water bodies; Panel C presents the normalized difference vegetation index (NDVI), which indicates vegetation coverage; and Panel D shows the normalized difference drought index (NDBSI), which reflects the island’s drought level.

### 2.2. Data Sources and Preprocessing

The remote sensing data employed in this study are sourced from the Landsat and Sentinel−2A satellite series. Landsat 5 and Landsat 8 imagery were used to monitor land use changes and coastline dynamics in 2002, 2007, 2013, and 2017, whereas Sentinel−2A imagery was used for monitoring in 2022 (Table 1). These data were all sourced from the Google Earth Engine (GEE) cloud platform (https://code.earthengine.google.com, accessed on 28 September 2024.) and underwent rigorous preprocessing, encompassing cloud removal, radiometric correction, and geometric correction, to ensure sufficient image quality and analysis accuracy.

**Figure 2:**
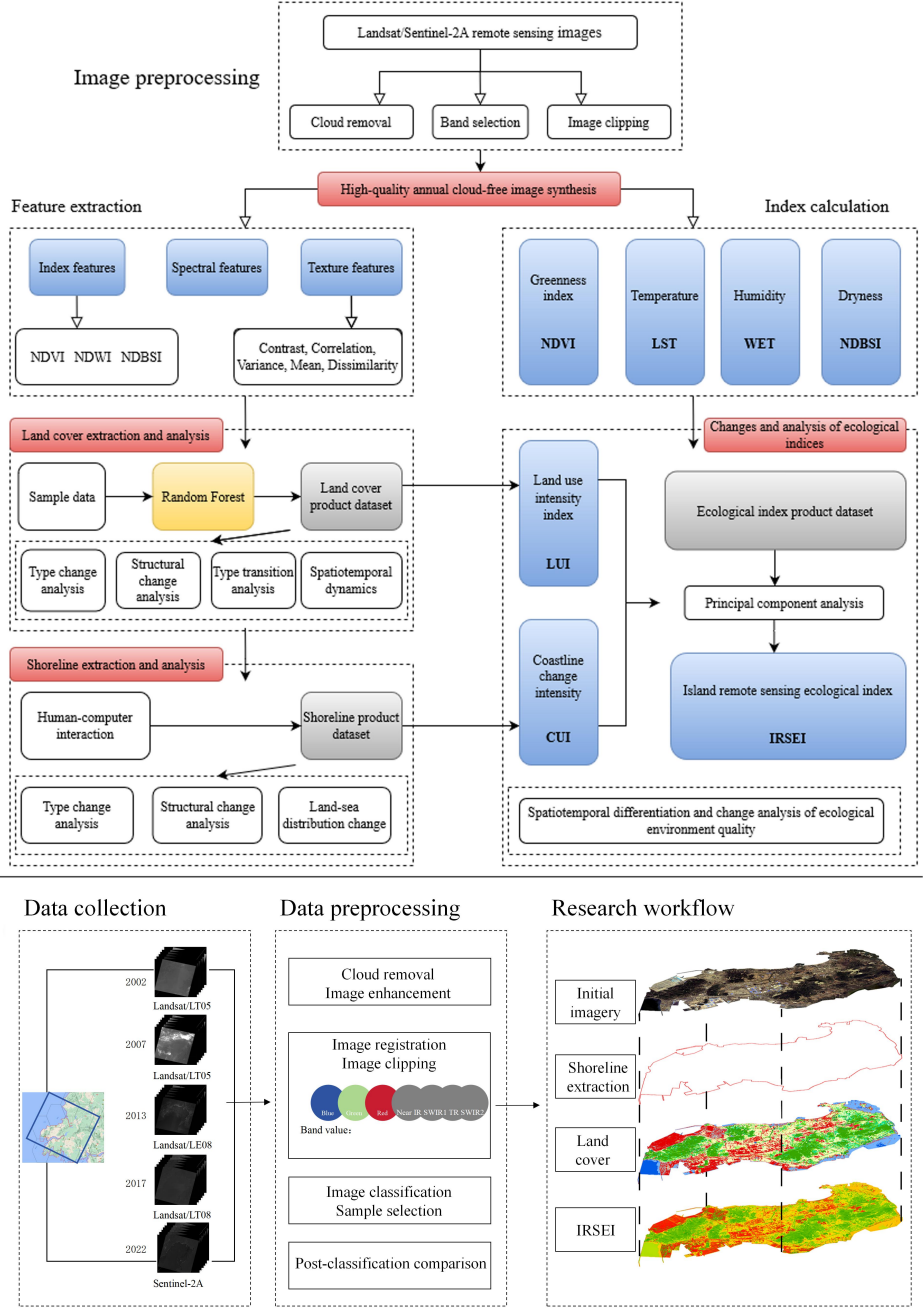
Technical flowchart of this study.

Image preprocessing involved radiometric correction, geometric alignment, and cloud removal. The “simpleComposite” algorithm provided on the GEE platform was applied to filter images with less than 10% cloud coverage to ensure data quality [32]. Furthermore, the images were cropped to the boundaries of the study area to obtain high−quality input data for the subsequent analyses of land use, coastline dynamics, and the ecological environment (Figure 2).

The Landsat satellite series has provided high temporal resolution remote sensing imagery since 1972, offering valuable data support for global environmental monitoring [33,34]. Sentinel−2A provides data with a high spatial resolution and frequency, making these data especially suitable for detailed regional analysis and vegetation monitoring. The integration of both satellites provides a robust foundation for long−term, multiscale, and multi−indicator environmental assessments [35,36].

### 2.3. Land Use Change Analysis

Using the GEE platform, multitemporal remote sensing imagery was combined to perform land cover classification and feature extraction for the study area [37]. The classification process involved the use of the random forest algorithm as a machine learning model to classify the study area on the basis of multispectral features in the remote sensing imagery [38].

To ensure the scientific validity and accuracy of the classification process, the land cover classification system (LCCS) proposed by the Food and Agriculture Organization (FAO) was used as the classification standard. Guided by this classification standard, the land cover types on Changxing Island were defined and categorized into the following eight classes (Table 2): cultivated land, forest, grassland, shrubland, aquaculture ponds, water bodies, artificial surfaces, and bare land.

For spectral feature extraction, a feature set was constructed for use with the random forest model. This process included extracting the normalized difference vegetation index (NDVI), normalized difference built−up index (NDBI), and normalized difference water index (NDWI) as feature variables via a multispectral image combination formula (Table 3).

#### Land Cover Classification and Accuracy Assessment

In this study, we used remote sensing imagery to classify the land cover on Changxing Island. To validate the accuracy of the classification results, the kappa coefficient was employed to assess the precision of the classification [39]. The kappa coefficient is a commonly used statistical measure applied to evaluate the consistency between two sets of classification results, especially for comparing remote sensing classification results with field observation data [40].

### 2.4. Shoreline Extraction and Change Analysis

To extract the shoreline, the cloud−free image composite module in the GEE was initially used to select typical images for the years 2002, 2007, 2013, 2017, and 2022 to ensure data consistency and high precision [41]. The adjusted water index (AWEI) [42] was subsequently calculated to distinguish water bodies from other surface features, and Otsu’s thresholding method was applied to automatically separate water and land, with a water mask generated for shoreline extraction [43].

Following coastline extraction, the “reduceToVectors” method was applied to convert the raster images into vector form. Geometric simplification and topological optimization were then used to further increase the accuracy of the extracted coastline. Next, the extracted coastline data were overlaid with multitemporal imagery, and metrics such as the coastline change intensity (CCI) (Table 4) were used to quantify the spatiotemporal changes in the coastline to reveal the long−term effects of reclamation activities and human intervention on coastal evolution [44,45].

### 2.5. Method for Evaluating Island Ecological Quality

In recent years, the remote sensing ecological index (RSEI) has been extensively applied for the comprehensive evaluation of environmental quality [46,47]. The RSEI integrates ecological parameters, such as vegetation cover, humidity, dryness, and surface temperature, to quantify changes in the ecological environment and directly reflect the influence of human activities on the environment [48,49]. Owing to the unique characteristics of island ecosystems, such as the limited land area, fragile ecological environment, and high dependence on human activities [50,51], the existing remote sensing ecological index (RSEI) may not fully account for the impact of certain factors on ecological quality. Therefore, developing a remote sensing ecological index specifically suited for island ecosystems (IRSEI) is particularly important. The IRSEI not only provides an accurate indication of changes in the ecological environment of islands but also encompasses the interactions among various ecological factors on islands, such as types of land use and vegetation cover, offering a scientific basis for ecological conservation and sustainable development on islands. This method is particularly suitable for large−scale remote sensing data analysis and assessments of regional ecological conditions and their dynamic evolution, and serves as an efficient basis for comprehensive evaluation of ecological quality [52,53]. Building upon the RSEI research framework, an improved remote sensing ecological index specifically for island regions, namely, the island remote sensing ecological index (IRSEI), is established in this study. The IRSEI builds upon the RSEI by incorporating more targeted ecological parameters, such as the NDVI, a humidity index (WET) [54], a dryness index (NDBSI) [55], a heat index (LST) [56], and a land use intensity index (LUI) [57]. These parameters collectively constitute a comprehensive and multidimensional indicator system specifically aimed at evaluating the complex ecological environmental quality of island regions; thus, this indicator effectively captures the characteristics of ecosystem changes under the dual influences of natural forces and human activities.

(1) $$\begin{array}{cccc} WET_{TM} & =0.03\rho_{BLUE}+0.22\rho_{GREEN} & +0.29\rho_{RED}+0.17\rho_{NIR} & -0.68\rho_{SWIR1}-0.58\rho_{SWIR2} \end{array}$$

(2) $$\begin{array}{cccc} WET_{OLl} & =0.16\rho_{BLUE}+0.20\rho_{GREEN} & +0.32\rho_{RED}+0.35\rho_{NlR} & -0.70\rho_{SWIR1}-0.45\rho_{SWIR2} \end{array}$$

(3) $$\begin{array}{cccc} WET_{S2} & =0.15\rho_{BLUE}+0.18\rho_{GREEN} & +0.34\rho_{RED}+0.33\rho_{NlR} & -0.72\rho_{SWIR1}-0.48\rho_{SWIR2} \end{array}$$

The humidity index (WET) is based on the wetness index obtained via the tasselled cap transformation, with adjusted weights to accommodate different sensors (Landsat TM, *OLI*, and Sentinel−2), while also emphasizing the differences in moisture contents among vegetation, soil, and water bodies. The new weights can be used to enhance the sensitivity to moisture levels in ecological monitoring. $\rho_{BLUE}$ represents the reflectance of the blue light band, $\rho_{GREEN}$ represents the reflectance of the green light band, $\rho_{RED}$ represents the reflectance of the red light band, $\rho_{NIR}$ represents the reflectance of the near−infrared band, $\rho_{SWIR1}$ represents the reflectance of the shortwave infrared 1 band, and $\rho_{SWIR2}$ represents the reflectance of the shortwave infrared 2 band.

(4) $$GNDVI=\frac{\rho_{\mathrm{NIR}}-\rho_{\mathrm{GREEN}}}{\rho_{\mathrm{NIR}}+\rho_{\mathrm{GREEN}}}$$

The modified Greenness Index (GNDVI), which is based on sensitivity to vegetation growth, encompasses the normalized difference between near−infrared and green light to enhance the sensitivity to vegetation growth. By substituting the green light band for the red light band, this index is responsive to the spectral characteristics of green leaves [58]. The bands used in this formula are consistent with those noted in the aforementioned formula.

(5) $$M-NDBSI=\frac{SI+IBI}{2+\sigma (SI,IBI)}$$

(6) $$SI=\frac{\rho_{\mathrm{SWIR1}}+\rho_{\mathrm{RED}}-\rho_{\mathrm{NIR}}-\rho_{\mathrm{BLUE}}}{\rho_{\mathrm{SWIR1}}+\rho_{\mathrm{RED}}+\rho_{\mathrm{NIR}}+\rho_{\mathrm{BLUE}}}$$

(7) $$IBI=\frac{\rho_{\mathrm{SWIR2}}+\rho_{\mathrm{SWIR1}}+\rho_{\mathrm{NIR}}}{\rho_{\mathrm{RED}}+\rho_{\mathrm{GREEN}}+\rho_{\mathrm{BLUE}}}$$

This approach allows for a more nuanced understanding of the variability in dryness by incorporating both the average condition and the distribution of bare soil and built−up areas. By incorporating the standard deviation, uncertainty is introduced, enhancing the detection of surface desiccation [59]. σ(SI,IBI) represents the standard deviations of SI and IBI. The bands used in this formula are consistent with those described in the aforementioned formula.

(8) $$TIR=\frac{LST-LST_{\min}}{LST_{\max}-LST_{\min}}$$

(9) L=gain×DN+bias

(10) $$T=\frac{K_{2}}{\ln\left(\frac{K_{1}}{L}+1\right)}$$

(11) $$LST=\frac{T}{1+\lambda \cdot \frac{T}{\rho }\cdot \ln\left(\varepsilon \right)}$$

The heat index uses a land surface temperature parameter, with further modifications to reduce the influence of the absolute temperature value on the model [60]. TIR represents the radiance at the sensor, DN represents the greyscale value of the image, *T* represents the sensor temperature, $K_{1}$ and $K_{2}$ are calibration coefficients, λ represents the wavelength, ϵ represents the surface emissivity, and $\rho =1.438\times 10^{-2}\;m.$

(12) $$LUI=\frac{\Sigma_{i=1}^{n}A_{i}\times W_{i}}{\sum_{i=1}^{n}A_{i}}$$

Inspired by the theory of land use intensity, the coastal development activity intensity index was designed to indicate the extent of human intervention along the coastline and the level of coastal utilization to quantitatively evaluate the disturbance level associated with various types of human development activities along the coast [61]. $A_{i}$ represents the area of land type *i*, and $W_{i}$ represents the weight of the corresponding land type (proportional to its utilization intensity). Bare land is classified as level 1; water bodies, forestland, grassland, and shrubland are classified as level 2; farmland and aquaculture ponds are classified as level 3; and construction land is classified as level 4.

During the calculation of the IRSEI, each ecological parameter is first normalized to eliminate scale differences among different parameters, ensuring that all the ecological parameters have equal weights in the IRSEI calculation. PCA is subsequently used to determine the contribution of each ecological parameter to the overall ecological quality assessment, and a comprehensive IRSEI model is ultimately constructed [62,63]. PCA is conducted on the five normalized ecological indicators to explore their temporal trends and summarize their impacts on the overall quality of the Changxing Island ecosystem. PCA is a commonly used dimensionality reduction technique in comprehensive ecological assessments and is capable of effectively revealing the correlations and trends among complex ecological variables [64]. Specifically, principal component analysis (PCA) allows us to assess the relative importance of each ecological indicator by analysing their loadings in relation to the principal components. The loadings represent the contributions of all variables to the principal components, which is crucial for evaluating the influence of individual ecological factors on overall ecosystem quality. This method of evaluating variable importance through loadings has been widely applied in ecological studies. In one study, loadings were calculated via PCA to reveal the contribution of each ecological indicator to the principal components. The loadings reflect the relative importance of each variable to the principal components, with a high loading indicating a high contribution for that variable [65]. Therefore, loadings are useful for identifying and quantifying the key factors influencing changes in an ecosystem. This model can be used to comprehensively quantify the spatial distribution and temporal changes in island ecological quality, revealing the dynamic evolution characteristics of environmental degradation and recovery.

(13) $$IRSEI=\frac{1}{5}\left(GNDVI+NDWI+\left(1-M-NDBSI)+(1-LST)+(1-LUI\right)\right)$$

*GNDVI*, *NDWI*, *M−NDBSI*, *LST*, and *LUI* need to be normalized to the range [0, 1].

Since the dimensions of the ecological indicators are different, they need to be normalized before performing principal component analysis, with all indicator values mapped to the range [0, 1]. The normalization formula for the indicators is as follows:

(14) $$I_{norm}\left(i\right)=\frac{I\left(i\right)-I_{min}}{I_{max}-I_{min}}$$

To ensure that the *IRSEI* values for different regions or years are consistent in scale and comparable, further normalization is performed:

(15) $$IRSEI=\frac{IRSEI_{0}-IRSEI_{min}}{IRSEI_{max}-IRSEI_{min}}$$

where $I_{norm}\left(i\right)$ represents the normalized indicator value at pixel *i*, I(i) denotes the original indicator value, and $I_{max}$ and $I_{min}$ represent the maximum and minimum values of the indicator, respectively. To avoid the impact of large water bodies on the evaluation of ecological quality, a water index is used to mask water areas. Within the unmasked area, six normalized ecological indicators are used for PCA. To ensure that a high PC1 value represents high ecological quality and to facilitate comparisons among different regions, a transformation is applied to the PC1 value. The final IRSEI range is [0, 1]. The better the ecological quality is, the closer the IRSEI value is to 1; conversely, the poorer the ecological quality is, the closer the IRSEI value is to 0. The specific formula is as follows:

(16) $$IRSEI_{0}=1-PC1(f\left(GNDVI,WET,M-NDBSI,LST,LUI\right)$$

where ${IRSEI}_{0}$ is the initially calculated ecological index and ${IRSEI}_{max}$ and ${IRSEI}_{min}$ represent the maximum and minimum values of this index, respectively.

## 3. Results

### 3.1. Overview of Land Use Changes on Changxing Island

An analysis of the remote sensing images of Changxing Island from 2002, 2007, 2013, 2017, and 2022 revealed that land use changed significantly. The farmland area decreased from 97.21 km^2^ in 2002 to 76.39 km^2^ in 2022, a reduction of 20.82 km^2^, with conversion mainly to construction land. During this period, construction land expanded from 8.89 km^2^ to 65.26 km^2^, an increase of 634.08%. The forest area decreased from 71.54 km^2^ to 58.90 km^2^, whereas the area of aquaculture ponds expanded from 15.43 km^2^ to 24.35 km^2^. The land use classification results at each time point are highly accurate, with overall classification accuracy exceeding 89% and kappa coefficients above 0.88.

#### 3.1.1. Evaluation of the Land Use Classification Accuracy

Using a random forest classifier, the land use on Changxing Island was classified at five time points, and the accuracy of the classification results was assessed (Table 5). By sampling 677 representative land use samples covering various land types, and cross−referencing them with historical satellite images from the GEE, the accuracy of the land use attributes was verified.

The results indicated that the overall classification accuracies for 2002, 2007, 2013, 2017, and 2022 were 91.07%, 90.43%, 89.75%, 90.23%, and 94.36%, respectively, with kappa coefficients all exceeding 0.88, suggesting that the classification model yields high overall prediction accuracy [66]. Moreover, the overall accuracy and kappa coefficient for 2022 were higher than those for 2002, 2007, 2013, and 2017, indicating that classification accuracy is largely influenced by the spatial resolution of the imagery selected.

#### 3.1.2. Area Changes in Land Use Types

**Figure 3:**
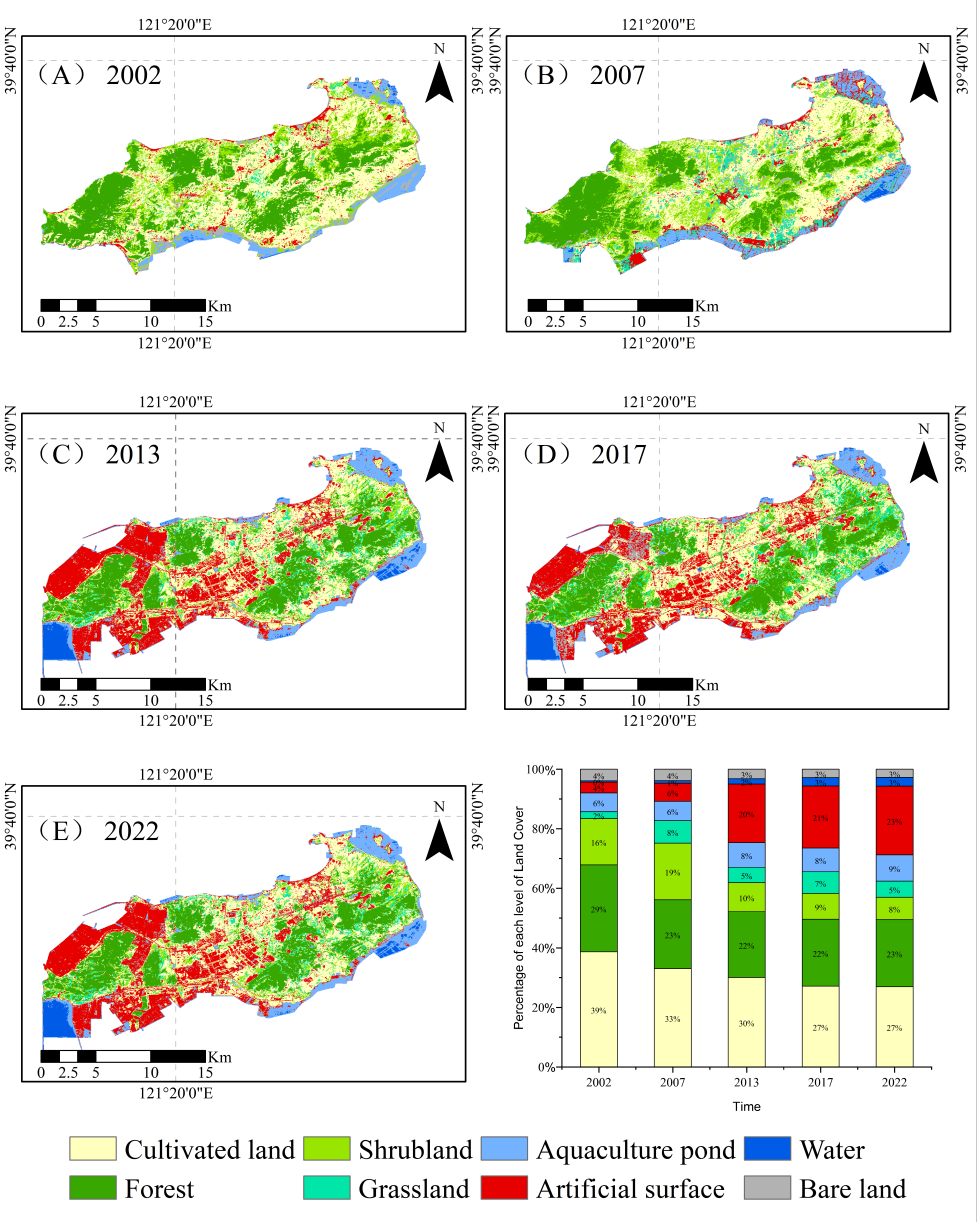
Spatial distribution of land use types on Changxing Island from 2002 to 2022. (**A**) Land use types of Changxing Island in 2002, (**B**) Land use types of Changxing Island in 2007, (**C**) Land use types of Changxing Island in 2013, (**D**) Land use types of Changxing Island in 2017, (**E**) Land use types of Changxing Island in 2022.

A systematic analysis of the changes in the areas of land use types on Changxing Island at five time points, namely, 2002, 2007, 2013, 2017, and 2022, was conducted. The results indicate that the land use structure of Changxing Island has changed significantly over the past 20 years, as primarily reflected in the changes in the areas of farmland, forestland, aquaculture ponds, and construction land (Figure 3).

Specifically, between 2002 and 2022, the farmland area decreased from 97.21 km^2^ to 76.39 km^2^, representing a decrease of 21.42%. The forest area also decreased from 71.54 km^2^ to 58.90 km^2^, a reduction of 17.67%. These changes reflect a trend of conversion and reduction in farmland and forest within the island area, mainly due to urban development and changes in land use practices. Moreover, areas of construction land have experienced significant growth. The expansion of construction land has been particularly significant, increasing from 8.89 km^2^ to 65.26 km^2^, an increase of 634.08%.

**Figure 4:**
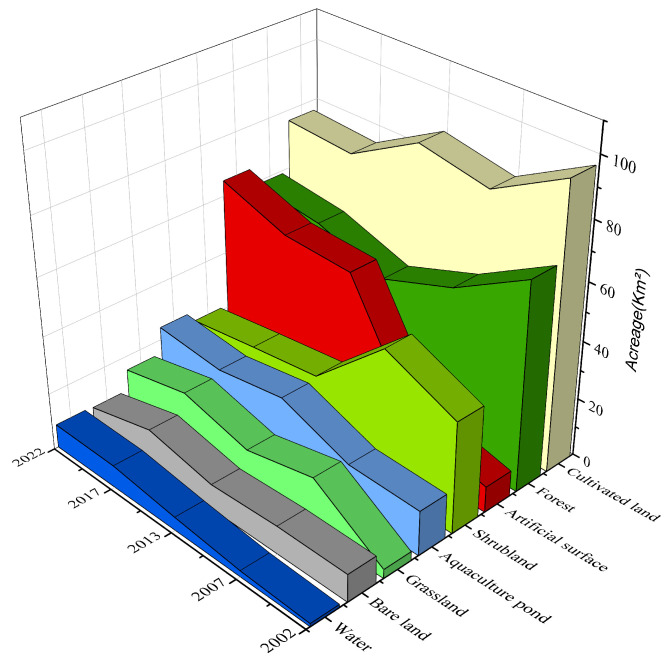
Spatial distribution of land cover changes on Changxing Island from 2002 to 2022.

Over the past 20 years, land use changes on Changxing Island have generally been marked by reductions in farmland and forest areas, alongside significant expansions of construction land and aquaculture ponds (Figure 4).

**Figure 5:**
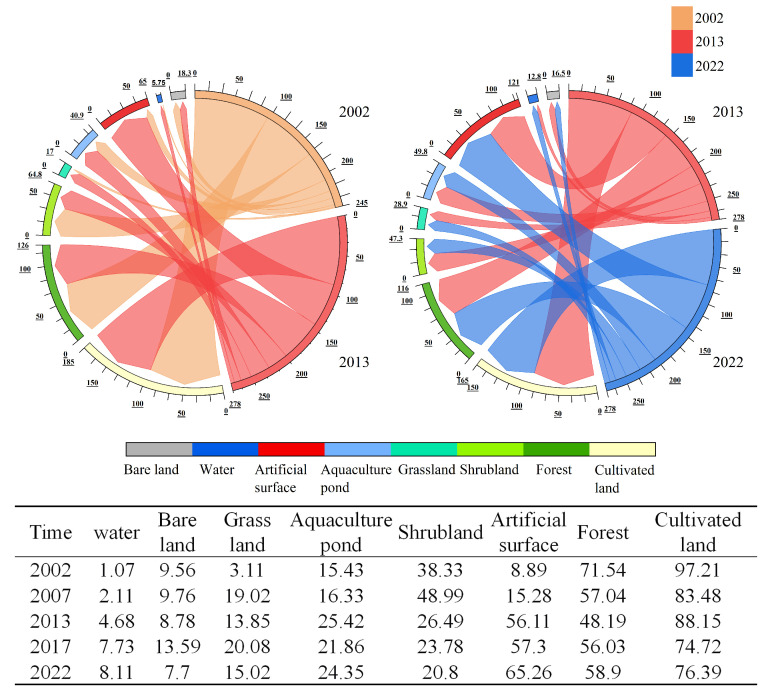
Spatial transfer map of land cover types and land cover datas on Changxing Island from 2002 to 2022.

The figure shows the transitions between different land use types on Changxing Island from 2002 to 2022 (Figure 4); the stacked effect illustrates the relationships between different land cover types and their changes over the years. From the figure, it is evident that the most prominent trend is the conversion of farmland into construction land, especially between 2002 and 2013, when the farmland area decreased significantly, mainly through conversion to construction land (Figure 5), where the width of the arcs represents the frequency of conversion between land types. The area changes for each land use type in Figure 5 correspond to the conversion relationships shown in Figure 4. Farmland and forest areas have significantly decreased, whereas construction land and aquaculture ponds have continued to expand.

### 3.2. Overview of Shoreline Changes on Changxing Island

The analysis results indicate that, over the past 20 years, Changxing Island’s shoreline length has experienced significant growth, gradually increasing from 101.82 km in 2002 to 144.75 km in 2022, representing a total growth of 42.93 km and an average annual growth rate of 2.15 km/year. This change was driven mainly by reclamation projects and development activities, particularly during the periods from 2007 to 2013 and from 2017 to 2022, when the shoreline growth rate was rapid, reflecting significant urban expansion and coastal development in the region.

#### Shoreline Extraction and Accuracy Assessment

**Figure 6:**
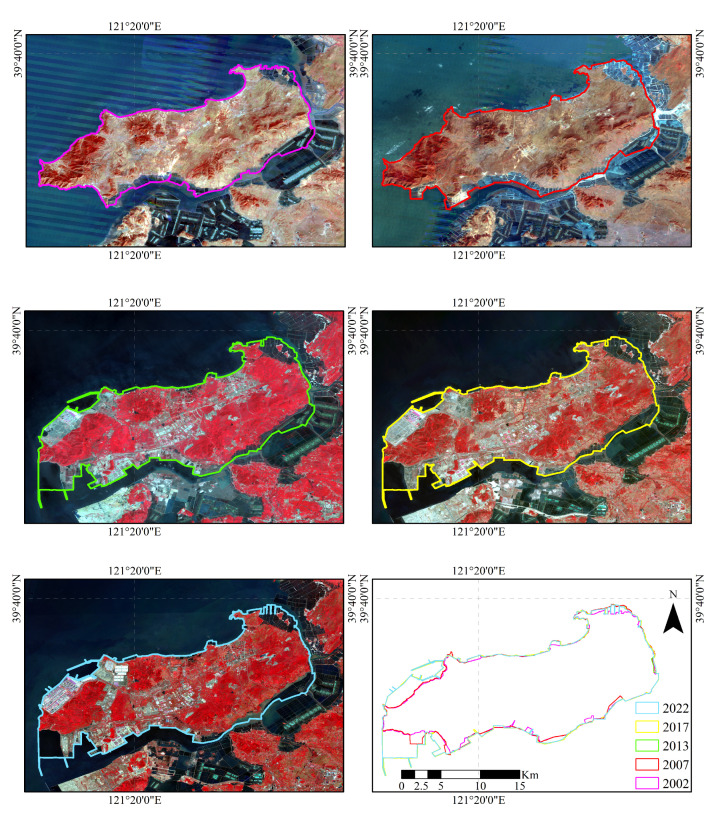
Images and coastline change maps of Changxing Island from 2002 to 2022.

According to the relevant formulas and analysis results, the length of the shoreline of Changxing Island exhibited a significant increasing trend between 2002 and 2022 (Figure 6).

From 101.82 km in 2002, the shoreline length increased to 106.28 km in 2007, then further to 127.37 km in 2013, reaching 131.89 km in 2017, and finally 144.75 km in 2022. Over the 20−year study period, the shoreline length increased by a total of 42.93 km, with an average annual growth rate of 2.15 km/year.

**Figure 7:**
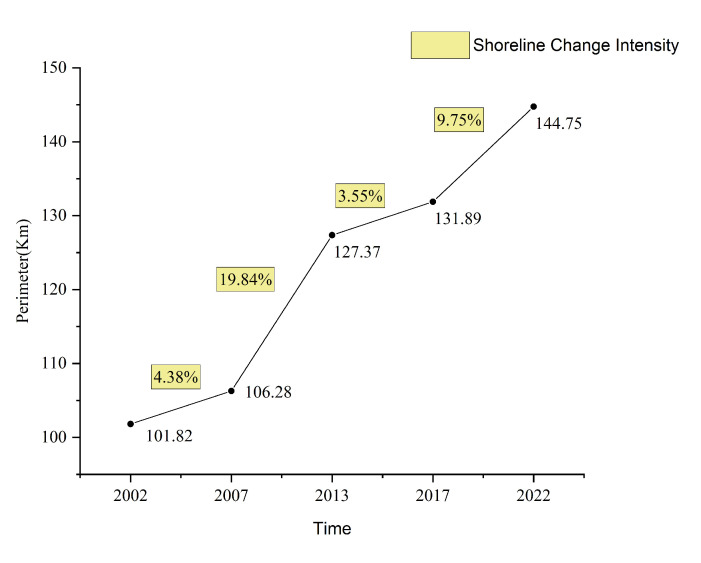
Coastline length variation and intensity of change on Changxing Island from 2002 to 2022.

Over time, the shoreline changes in Changxing Island can be divided into several phases, with the period from 2007 to 2013 corresponding to the most significant increase in shoreline length, with an increase of 21.09 km, an average annual growth rate of 4.22 km/year, and an intensity of change of 19.84%. From 2017–2022, the shoreline length increased by 12.86 km, with an average annual growth rate of 2.57 km/year and a change intensity of 9.75%, indicating a slowdown compared with the trends in previous phases. Correspondingly, the differences between these phases were significant, particularly from 2002 to 2007 and 2013 to 2017, when the increases were 4.46 km and 4.52 km, with change intensities of 4.38% and 3.55%, respectively, indicating significant differences in the scale and rate of reclamation at different times (Figure 7).

From a spatial perspective, the shoreline changes on Changxing Island were mainly concentrated in the reclaimed areas, especially during the peak development periods of 2007 to 2013 and 2017 to 2022. This led to notable shoreline changes, resulting in the expansion of facilities such as ports and aquaculture ponds, making the shoreline more regular and straight.

### 3.3. Overview of Ecological Index Changes on Changxing Island

In this section, a summary analysis of the trends of the IRSEI of Changxing Island is presented to assess the dynamic changes in ecological quality from 2002 to 2022. The IRSEI integrates the NDVI, WET, LST, NDBSI, and the LUI to reflect ecosystem health and the impact of human activities.

The analysis results indicated that the ecological quality of Changxing Island has significantly deteriorated over the past 20 years. The area of regions with good and excellent ecological quality decreased from 105.25 km^2^ in 2002 to 57.58 km^2^ in 2022, whereas the areas classified as fair and poor increased significantly to 32.51 km^2^ and 186.07 km^2^, respectively.

#### Temporally Dynamic Analysis of Ecological Index Changes

Using data from 2002, 2007, 2013, 2017, and 2022, PCA was performed for the five components of the Changxing Island ecological index (LST, LUI, M−NDBSI, GNDVI, and WET) (Table 6), and the first principal component in each year was determined.

Further analysis of the eigenvalues reveals that the IRSEI of Changxing Island exhibits some volatility and phase changes over time. Overall, the IRSEI rose between 2002 and 2007, from 0.4470 to 0.5185, suggesting that the ecological quality improved during this period. However, between 2007 and 2013, the average IRSEI decreased to 0.4338, a decrease of 0.0847, indicating a clear ecological degradation process. This degradation was likely related to the sharp increase in shoreline length and the expansion of human activities, particularly through the establishment of construction land and land reclamation activities, which intensified disturbances to the natural ecology. During the period from 2017 to 2022, the IRSEI recovered, gradually increasing to 0.4768, indicating restorative improvement in the overall ecological environment following the implementation of ecological restoration measures in some areas in recent years.

**Figure 8:**
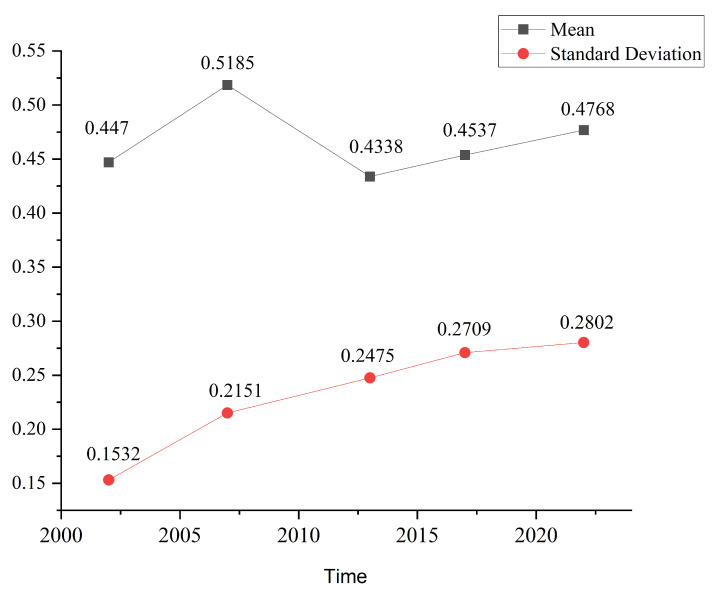
Changes in the mean and standard deviation of the IRSEI for Changxing Island from 2002 to 2022.

Overall, the IRSEI increased cumulatively by 0.0298 over the 20−year study period (Figure 8), suggesting that, while the ecological quality has experienced fluctuations, it has improved in recent years. The ecological quality has remained stable, except changes due to the increase in construction land, and no other significant changes have been observed.

**Figure 9:**
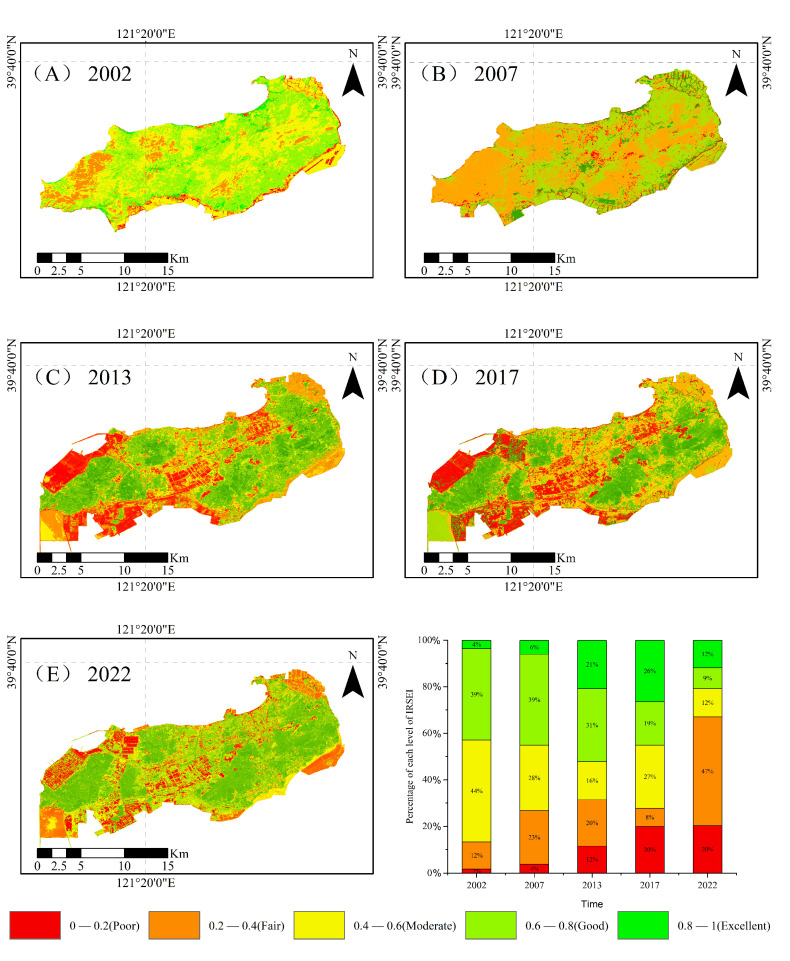
Spatial distribution of the environmental quality levels on Changxing Island from 2002 to 2022. (**A**): Environmental quality level of Changxing Island in 2002, (**B**): Environmental quality level of Changxing Island in 2007, (**C**): Environmental quality level of Changxing Island in 2013, (**D**): Environmental quality level of Changxing Island in 2017, (**E**): Environmental quality level of Changxing Island in 2022.

To further assess the spatial distribution characteristics of ecological quality at different points in time in each region, we constructed IRSEI spatial distribution maps for different periods along with changes in its five levels (Figure 9) and calculated the area proportions of the five levels in different years. This approach offers a detailed temporal and spatial perspective on the ecological quality of Changxing Island, illustrating how ecological grades evolve across different regions over time.

**Figure 10:**
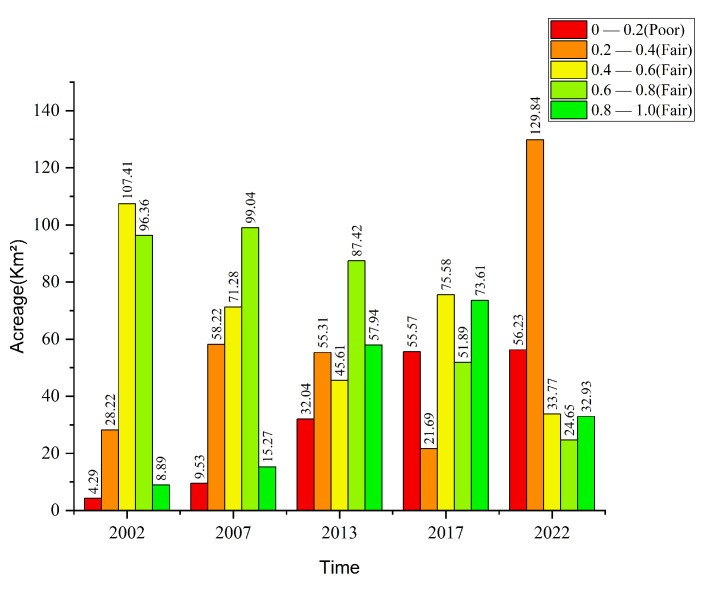
Proportions of ecological quality levels for Changxing Island from 2002 to 2022.

The overall quality of the ecological environment experienced significant degradation from 2002 to 2022 (Figure 10). In 2002, the area of good−quality areas on Changxing Island was 96.36 km^2^ (39.3%), and that of excellent−quality areas was 8.89 km^2^ (3.63%), totalling 105.25 km^2^ (42.93%); by 2022, the proportion of good−quality areas had decreased to 24.65 km^2^ (8.89%) and the proportion of excellent−quality areas had increased to 32.93 km^2^ (11.87%), totalling 57.58 km^2^ (20.76%). Moreover, the fair−quality and poor−quality areas increased significantly. In 2002, the area of poor−quality areas was 4.29 km^2^ (1.75%) and fair−quality was 28.22 km^2^ (11.51%); by 2022, the fair−quality area reached 129.84 km^2^ (46.78%) and that of poor−quality areas had increased to 56.23 km^2^ (20.27%), marking the highest value on record. The area of moderate ecological quality also decreased from 107.41 km^2^ (43.79%) in 2002 to 33.77 km^2^ (12.17%) in 2022, indicating the gradual degradation of moderately high−quality land. Overall, the area of high−quality ecological land significantly diminished, whereas those of poor and very poor land increased substantially, indicating the continuing deterioration of the ecological environment.

## 4. Discussion

### 4.1. Effects of Land Cover on the Island Remote Sensing Ecological Index (IRSEI)

**Figure 11:**
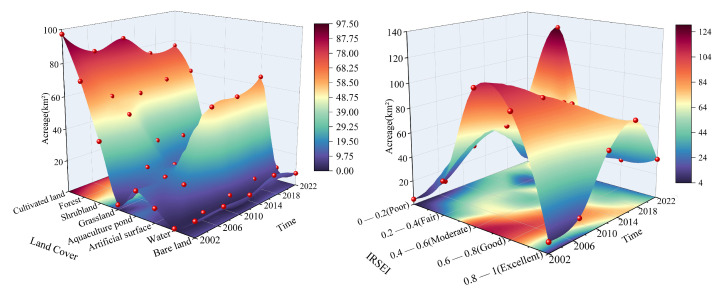
Spatiotemporal changes in the area intensity of land cover types on Changxing Island from 2002 to 2022.

Land cover changes caused by reclamation and urban expansion are key factors influencing IRSEI fluctuations. Like Changxing Island, other coastal islands in China have also experienced urban expansion [67,68]. Previous studies have also highlighted that urban expansion is a major driver of land cover changes [8]. Our research revealed that, from 2002 to 2022, the built−up area on Changxing Island increased by 83%, with the majority of this growth resulting from land reclamation projects. During urban expansion, other land use types (such as cultivated land and forest) tend to shift towards built−up land. As a result, the IRSEI value declined in the short term, but it slowly recovered because of the development of green infrastructure within the urban areas (Figure 11).

### 4.2. Effects of Shoreline Changes on the Island Remote Sensing Ecological Index (IRSEI)

From 2002 to 2022, the shoreline perimeter of Changxing Island increased from 101.82 to 144.75 km, with significant reclamation activities and shoreline development in Bohai Bay [69]. On Changxing Island, the integrity of the ecosystem has been significantly affected by the intensification of reclamation projects and the development of the coast. Artificial land reclamation often destroys existing wetland and coastal ecosystems, which are typically important providers of ecosystem services such as water purification, carbon sequestration, and biodiversity conservation [70,71]. Therefore, changes in the coast directly affect the IRSEI evaluation results. Our study revealed that changes on the coast of Changxing Island significantly affect the IRSEI fluctuations. Particularly in the early stages of reclamation and shoreline development, the IRSEI value decreased in the short term because of human activities and the reduction in natural wetlands [72]. However, as ecological compensation measures were implemented in shoreline development projects (such as the construction of artificial wetlands and ecological green spaces), the ecological environment gradually recovered, and the IRSEI value partially recovered. This suggests that although shoreline changes can lead to a short−term decline in ecological quality, the construction of green infrastructure can effectively promote ecological restoration and improve the IRSEI.

**Figure 12:**
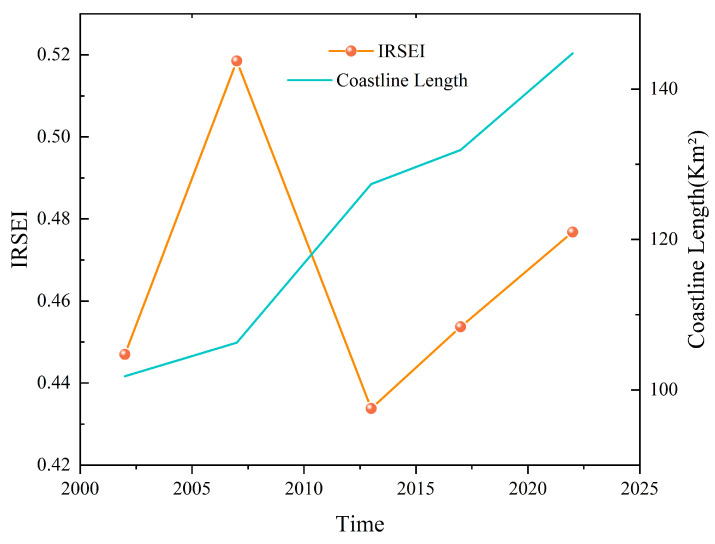
Relationships between coastline length and changes in the IRSEI of Changxing Island from 2002 to 2022.

This result is generally consistent with those of the above studies, indicating that artificial changes in the shoreline can have a profound impact on the ecological quality of an island [72]. Therefore, future research could focus on the long−term effects of different types of shoreline development on ecological quality, and the effectiveness of ecological compensation measures in restoring the ecological environment could be evaluated (Figure 12).

### 4.3. Limitations and Future Prospects

The spatial and temporal resolutions of remote sensing data limit the ability to capture small−scale ecological changes in many areas. Although high−resolution remote sensing imagery was used in this study, certain small−scale ecological changes (such as the construction of urban green spaces and the restoration of local wetlands) may not have been fully reflected [73,74,75]. Furthermore, the temporal resolution of remote sensing imagery is relatively low, which can prevent the capture of rapidly changing ecological events and short−term environmental fluctuations. Therefore, future research should integrate high−frequency remote sensing data or field surveys to further improve the accuracy of analyses. Additionally, the quality of the data may introduce some uncertainty. Although we applied various data correction methods (such as atmospheric correction and geometric correction), remote sensing data may still be affected by factors such as cloud cover, atmospheric conditions, and sensor accuracy. Although these impacts are minimized through the standard processing workflow on the GEE platform, remaining errors may still influence the final IRSEI assessment results.

Future research could focus on enhancing the ability to capture small−scale ecological changes by integrating high−resolution remote sensing data with multisource data fusion. The use of these high−precision data, in conjunction with advanced technologies such as machine learning [76,77], will improve the accuracy and comprehensiveness of ecological quality assessments, further advancing ecological monitoring and sustainable development research.

## 5. Conclusions

In this study, we used advanced remote sensing technology along with the Google Earth Engine (GEE) platform to analyse the spatiotemporal evolution characteristics of the ecological environment on Changxing Island, Dalian, China, from 2002 to 2022. By processing Landsat and Sentinel−2A remote sensing data, the random forest classification algorithm and a postclassification comparison method were applied to accurately extract land cover types and shoreline changes. Additionally, the Integrated Remote Sensing Ecological Index (IRSEI) model was developed to evaluate ecological quality, incorporating parameters such as the vegetation index, wetness index, dryness index, heat index, and land use intensity. This study yielded the following conclusions:

1. Changxing Island experienced significant changes in land use and shoreline expansion from 2002 to 2022. Farmland decreased substantially, while construction land increased notably, indicating rapid urbanization. The total shoreline length increased continuously over the 20−year period, with an average annual increase of 2.15 km, primarily driven by reclamation and coastal development. The quality of the ecological environment declined significantly, with high−quality areas decreasing annually, while areas classified as poor and very poor expanded continuously, indicating that the health of the ecosystem is facing serious degradation issues.
2. The use of multisource data and cloud computing platforms enabled an in−depth analysis of the dynamic characteristics of the ecological environment on Changxing Island. The advantages of large−scale remote sensing data processing methods were highlighted, providing scientific support for local governments in developing ecological protection and sustainable development strategies. However, it is essential to integrate socioeconomic factors further to fully understand the multidimensional impacts of human activities on the ecological environment.

## Figures and Tables

**Table 1:** Series of Landsat and Sentinel images of Changxing Island from 2002 to 2022.

Sensors	Data	Spatial Resolution	Acquisition Date
Landsat/LT05	LANDSAT/LT05/ C02/T1_TOA	30 m	May 2002/May 2007
Landsat/LC08	LANDSAT/LC08/ C02/T1_L2	30 m	May 2013/May 2017
Sentinel−2A	COPERNICUS/ S2_SR_HARMONIZED	30 m	May 2022

**Table 2:** Land cover classification.

Land Class	Definition
Cultivated land	Land used for agricultural planting, such as the cultivation of grains, cash crops, and vegetables, as a fundamental resource for agricultural production.
Forest	Land typically found in mountainous, hilly, and similar regions.
Water	Land covered by water, primarily consisting of rivers, lakes, seas, and other water bodies.
Grassland	Grassland refers to vegetated areas mainly composed of herbaceous plants, encompassing both natural and cultivated grasslands.
Shrubland	Vegetated areas typically found in arid regions, mainly composed of mountain shrubs, evergreen shrubs, and similar vegetation types.
Bare land	Includes natural lands like sandy beaches, gravel areas, and bare rock−covered land, with minimal vegetation cover.
Aquaculture pond	Artificial or naturally constructed ponds used for aquaculture.
Artificial surface	Land characterized by human development activities, such as urban residential areas and infrastructure land.

**Table 3:** Formulas and explanations of NDVI, NDWI, and NDBI.

Index	Formula	Explanation
NDVI	$NDVI=\frac{L_{\mathrm{NIR}}-L_{\mathrm{RED}}}{L_{\mathrm{NIR}}+L_{\mathrm{RED}}}$	$L_{NIR}$ and $L_{RED}$ correspond to the near−infrared and red bands of Landsat imagery, respectively [36]
NDBI	$NDBI=\frac{L_{\mathrm{MIR}}-L_{\mathrm{NIR}}}{L_{\mathrm{MIR}}+L_{\mathrm{NIR}}}$	$L_{NIR}$ and $L_{MIR}$ represent the red and green bands of Landsat imagery, respectively [37]
NDWI	$NDWI=\frac{L_{\mathrm{GREEN}}-L_{\mathrm{NIR}}}{L_{\mathrm{GREEN}}+L_{\mathrm{NIR}}}$	$L_{NIR}$ and $L_{GREEN}$ correspond to the near−infrared and green bands of Landsat imagery, respectively [38]

**Table 4:** AWEI and CCI formulas and explanations.

Index	Formula	Explanation
*AWEI*	$\begin{array}{cc} AWEI & =4\times (GREEN-SWIR1)\\ & -(0.25\times NIR\\ & +2.75\times SWIR2) \end{array}$	GREEN corresponds to Band 3 in Landsat imagery and Band 3 in Sentinel−2 imagery. SWIR corresponds to Band 6 in Landsat imagery and Band 11 in Sentinel-2 imagery. NIR corresponds to Band 5 in Landsat imagery and Band 8 in Sentinel−2 imagery. SWIR2 corresponds to Band 7 in Landsat imagery and Band 12 in Sentinel−2 imagery.
*CCI*	$CCI=\frac{L_{t2}-L_{t1}}{T}$	$L_{t1}$ and $L_{t2}$ represent the shoreline lengths at times $t_{1}$ and $t_{2}$ (in km), respectively, and *T* represents the time interval (in years).

**Table 5:** Evaluation of surface cover type accuracy.

Time	Overall Classification Accuracy/%	Kappa Coefficient
2002	91.0714%	0.8952
2007	90.4255%	0.8877
2013	89.7540%	0.8805
2017	90.2255%	0.8815
2022	94.3625%	0.9271

**Table 6:** Principal component analysis of key indicators for Changxing Island from 2002 to 2022.

Time	LST	LUI	M−NDBSI	GNDVI	WET	Total Eigenvalue Sum
2022	0.0405	0.7762	0.4498	−0.4316	−0.0848	0.1840
2017	0.0548	0.8833	0.3138	−0.3349	−0.0781	0.1544
2013	0.1777	0.6530	0.5002	−0.5278	−0.1136	0.1878
2007	−0.0046	−0.9981	−0.0262	0.0538	−0.0121	0.0682
2002	−0.1138	−0.8327	−0.3884	0.0359	0.3752	0.1012

## Data Availability

The datasets generated during the study are available from the corre sponding author upon reasonable request.
